# Harnessing physical activity monitoring and digital biomarkers of frailty from pendant based wearables to predict chemotherapy resilience in veterans with cancer

**DOI:** 10.1038/s41598-024-53025-z

**Published:** 2024-01-31

**Authors:** Gozde Cay, Yvonne H. Sada, Mohammad Dehghan Rouzi, Md Moin Uddin Atique, Naima Rodriguez, Mehrnaz Azarian, M. G. Finco, Sarvari Yellapragada, Bijan Najafi

**Affiliations:** 1 Digital Health and Access Center (DiHAC), Michael E. DeBakey Department of Surgery, Baylor College of Medicine, Houston, TX USA; 2 Michael E. DeBakey Department of Veterans Affairs Medical Center, Houston, TX 77030 USA

**Keywords:** Cancer, Predictive markers, Biomedical engineering

## Abstract

This study evaluated the use of pendant-based wearables for monitoring digital biomarkers of frailty in predicting chemotherapy resilience among 27 veteran cancer patients (average age: 64.6 ± 13.4 years), undergoing bi-weekly chemotherapy. Immediately following their first day of chemotherapy cycle, participants wore a water-resistant pendant sensor for 14 days. This device tracked frailty markers like cadence (slowness), daily steps (inactivity), postural transitions (weakness), and metrics such as longest walk duration and energy expenditure (exhaustion). Participants were divided into resilient and non-resilient groups based on adverse events within 6 months post-chemotherapy, including dose reduction, treatment discontinuation, unplanned hospitalization, or death. A Chemotherapy-Resilience-Index (CRI) ranging from 0 to 1, where higher values indicate poorer resilience, was developed using regression analysis. It combined physical activity data with baseline Eastern Cooperative Oncology Group (ECOG) assessments. The protocol showed a 97% feasibility rate, with sensor metrics effectively differentiating between groups as early as day 6 post-therapy. The CRI, calculated using data up to day 6 and baseline ECOG, significantly distinguished resilient (CRI = 0.2 ± 0.27) from non-resilient (CRI = 0.7 ± 0.26) groups (p < 0.001, Cohen’s d = 1.67). This confirms the potential of remote monitoring systems in tracking post-chemotherapy functional capacity changes and aiding early non-resilience detection, subject to validation in larger studies.

## Introduction

Cancer is a major health concern in the United States, with approximately 1.9 million new cases projected in 2022, including 50,000 new cases within the Veteran’s Healthcare Administration^1^. Chemotherapy is a commonly used cancer treatment strategy, but its cytotoxic agents can have cancer-therapy related toxicity (CRT) side effects, such as fatigue, pain, nausea, neuropathy, anxiety, depression, or insomnia, which are estimated to affect approximately 86 to 88% of cancer patients^2,3^. Severe chemotherapy-induced toxicities may result in dose delay or dose reduction, chemotherapy discontinuation^4^, and unplanned healthcare service to manage these side effects^5^; some may even result in premature death, if left unmanaged^6,7^.

Resilience, often envisioned as the human capacity to maintain or regain relative stability in both psychological and physical realms amidst life's adversities, emerges as an integral concept within the sphere of cancer care^8,9^. The capability to assess and prognosticate this resilience in response to therapy-induced toxicity holds profound implications^9–11^. It directs both clinicians and patients through the maze of treatment decisions, offering an essential tool for customizing treatment plans to patient-specific responses. By anticipating potential adverse events, we may refine care strategies and explore alternative treatments, advancing personalized medicine. This predictive ability not only has clinical implications; it directly impacts patients' quality of life. By forecasting the heavy toll of side effects associated with cancer therapies, we may better navigate a path that manages these events, aiming to improve patient experiences. The early detection of toxicity offers a window for timely intervention, curtailing side effects and fostering improved outcomes^12^. The financial implications are also evident, as preparing for potential side effects can avert costly hospital readmissions and additional treatments^13^. Focusing on early stages of toxicity exposure is pivotal, allowing swift interventions that enhance patient outcomes and sustain quality of life^9^.

In the routine clinical practice of predicting therapy-related toxicity resilience, the performance status (PS) scales—Eastern Cooperative Oncology Group (ECOG) PS^14^ and Karnofsky Performance Status (KPS)^15^—stand as the current gold standards. These tools provide critical navigation through the complex decision-making process inherent in cancer treatment. While ECOG-PS exhibits strong correlations with patient quality of life and survival in clinical trials^16^ potential inaccuracies in broader PS assessments could unintentionally elevate treatment toxicity risk, leading to adverse clinical outcomes. The ECOG and KPS scales, drawing on self-reported patient data about daily activity levels and independence in performing essential tasks, are not devoid of inherent biases. Patient recall, preferred treatments of patients or providers, and inter-observer variability^17^ can skew evaluations. Furthermore, time constraints can impede the thoroughness of these assessments. Providers may not routinely revisit performance status post-initial consultation unless significant functional changes occur, or might opt for an "eyeball test," relying on observed patient movements instead of a comprehensive performance status history. Consequently, to discern patients with the highest likelihood of resilience to chemotherapy, it becomes imperative to investigate alternative, objective measures of functional status that can predict treatment-related toxicity and healthcare utilization.

With the emerging need for remote monitoring of digital biomarkers to predict therapy adverse events, this approach presents a timely and practical patient monitoring solution, which may potentially help enhancing therapy safety, and potentially reducing health disparities, particularly in rural settings where hospital access may be limited. Technological advances, especially in physical activity monitors, offer innovative avenues for functional status assessment. The value of remote monitoring using digital health tools was underscored during the COVID-19 pandemic^18–20^. These monitors can persistently gather objective digital biomarkers of mobility, such as lying or sitting position, daily steps, and gait speed, outside the clinical environment^21–27^. Given the relevance of ambulation and time spent in a sitting or lying position to ECOG-PS scoring, these mobility digital biomarkers could enrich PS assessment. Research has suggested the efficacy of physical activity monitors in gauging chemotherapy-induced peripheral neuropathy, cancer-related fatigue, and the level of fall concerns among older cancer survivors^28–30^. Wearable activity sensors also demonstrated good potential for tracking mobility performance in chemotherapy patients^31^ and showed significant correlation with ECOG-PS-collected performance status^32^.

In this study, we strive to assess the feasibility, acceptability, and proof-of-concept validity of a pendant physical activity monitor (PAMSys™, BioSensics LLC, MA, USA), a device with clinical validation for its ability to monitor cumulative postures such as sitting, standing, lying, and walking, along with tracking walking patterns, cadence, and postural transitions^33–36^. Our primary objective is to use this device to monitor patients' daily physical activities continuously throughout the first two weeks of therapy initiation, aiming to predict non-resilience to cancer toxicity-based therapy. This non-resilience is identified by events such as therapy discontinuation or dose reduction, cancer-related hospitalization within the first four weeks of therapy initiation, or death up to six months post-therapy. We hypothesize that real-time tracking of mobility-related digital frailty biomarkers, including slowness, weakness, fatigue, and inactivity^24^, from treatment onset could provide a more accurate prediction of adverse events compared to traditional assessments like the ECOG-PS. This research represents an important step in enhancing our understanding and application of wearable technology in oncology, with the potential to improve patient outcomes and personalize cancer treatment strategies.

## Methods

### Participants

We recruited Veterans with a recent diagnosis of stage IV lung or gastrointestinal cancer during their first chemotherapy infusion at the Michael E. DeBakey Veterans Affairs Medical Center (MDVAMC), Houston, Texas. All Veterans were > 18 years of age and were able to walk at least 15 feet independently, with or without an assistive device. We excluded Veterans who were not ambulatory, had no metastatic cancer, or refused to participate. All participants signed written informed consent forms. The Baylor College of Medicine Institutional Review Board and the Michael E. DeBakey Veterans Affairs Medical Center Research and Development Committee approved this study (IRB # H-48118) and all methods were performed in accordance with the relevant guidelines and regulations.

Demographic, medical history, baseline ECOG-PS, cancer diagnosis and treatment regimen of the participants were collected through chart review by a trained research coordinator. ECOG-PS was classified as dichotomous (0 or 1–2) for analysis (provided in Supplementary File). Hospital admissions and chemotherapy delays or dose reductions were also recorded. Participants completed psychosocial surveys for evaluation of their emotional, functional, and cognitive states before and after the intervention. For this purpose, we used the Center for Epidemiologic Studies Depression Scale (CES-D), Functional Assessment of Cancer Therapy-General (FACT-G), and the Mini-Mental State Examination (MMSE), respectively.

### Assessing frailty through mobility-driven digital biomarkers via a pendant sensor

Participants were instructed to continuously wear a water-resistant pendant sensor (PAMSys™, BioSensics LLC, Newton, MA, USA) around their neck for 14 days immediately following their first day of chemotherapy session, ensuring 24/7 wear. The monitoring period began with the onset of their initial chemotherapy and concluded at their next session. Additionally, participants were provided with a pre-stamped, pre-paid envelope and given clear instructions to return the sensor using this envelope at the end of the monitoring period.

**Figure 1:**
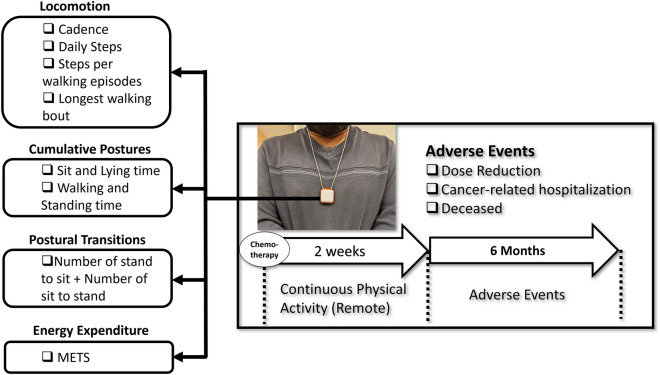
A patient wearing the PAMsys pedant sensor. Using validated algorithms different digital biomarkers of mobility including locomotion, cumulative postures, postural transitions, energy expenditure were extracted from the pendant sensor during a 2-week remote monitoring period (24h/7days) during chemotherapy.

The PAMSys™ (Fig. 1) is compact (3.5 cm × 3.5 cm × 1.5 cm) and lightweight (24g), equipped with a tri-axial accelerometer. It samples accelerometer data at a rate of 50Hz. Through validated algorithms and biomechanical models, it extracts various physical activity metrics^25,34–40^. These include cumulative postures (sitting, standing, lying, walking) with a time resolution of every second, locomotion metrics (e.g., cadence, step count, longest walking bout), postural transitions (e.g., number of sit-to-stand and stand-to-sit movements), and energy expenditure. In this study, we used the 2019 model of the PAMSys, which is water-resistant, has a battery life exceeding one month, and includes built-in data storage to ensure uninterrupted monitoring. We limited the recording to a minimum of 14 days of continuous monitoring and advised participants to return the sensor to the clinic. Alternatively, the sensor was collected when they came for their next cycle of chemotherapy. If a subject used the device for more than 14 days, our data analysis was confined to the first 14 days of recorded data. We selected a 14-day duration as it aligns with the bi-weekly chemotherapy cycle, allowing us to stop recording before the next cycle begins. This approach enables the tracking of changes in physical activity patterns immediately following the initiation of the first chemotherapy cycle and prior to the start of the subsequent cycle.

This study focuses on tracking changes in physical activity patterns from a defined baseline to a subsequent endpoint, using these patterns as surrogate markers for physical frailty phenotypes. Drawing upon previous research^24^, we selected nine frailty-associated digital biomarker features, as detailed in Table 1, to encapsulate these phenotypes. These features encompass dimensions such as slowness (indicated by cadence), inactivity (denoted by daily walking steps), weakness (depicted by postural transitions), and exhaustion (represented by factors like the duration of the longest walk, standing posture, and energy expenditure). Each of these biomarker features was assessed every 24 h, and their variations were tracked throughout the 14-day monitoring period.

**Table 1:** Sensor-derived digital frailty biomarkers.

Sensor-derived feature	Description	Phenotype
Walking cadence	Number of steps per minute in walking, 90th percentile	Slowness/weakness
Number of stand-to-sit	Postural transitions from a standing position to a sitting position	Slowness/weakness
Number of sit-to-stand	Postural transitions from a sitting position to a standing position	Slowness/weakness
Longest walking bout	Number of steps for longest unbroken walking	Exhaustion
Walking steps	Number of total walking steps	Inactivity
% of sitting	Percentage of sitting time for 24 h	Inactivity
% of standing	Percentage of standing time for 24 h	Inactivity
% of walking	Percentage of walking time for 24 h	Inactivity
% of lying	Percentage of lying time for 24 h	Inactivity

In terms of defining our time points, the baseline is considered as the first day of chemotherapy cycle. The endpoint is determined as final day of sensor monitoring (14th day). To capture the trajectory of each frailty biomarker, we calculated the ratio of the difference between the endpoint and baseline to the baseline itself. This computation provides a holistic depiction of the alterations in frailty over the course of the treatment period.

### Determination of chemotherapy resilience and chemotherapy resilience index

The recording of prospective adverse events was carried out via comprehensive reviews of medical charts, consultations with administering oncologists, and/or through interviews conducted either with the chemotherapy administering oncologists or directly with the participants themselves. The medical charts were collected through the electronic health record (EHR) within the Veterans Affairs (VA). This process led to the categorization of participants into one of two distinct groups for further analysis: resilient or non-resilient. The non-resilient classification was assigned based on the occurrence of certain prospective events related to cancer therapy. These included the discontinuation or dose reduction of therapy, cancer-related hospitalizations within the initial four weeks following therapy initiation, or mortality within a period of six months post-therapy. Conversely, resilient participants were defined as those who did not meet these criteria.

A two-sample t-test was used to compare categorical variables between the resilient and non-resilient groups. Cohen's d was used to calculate the effect size (small effect size (d = 0.2), medium effect size (d = 0.5), and large effect size (d = 0.8))^41^.

Three different models using Logistic Regression were tested to assess their ability to differentiate between resilient and non-resilient participants. The models included: (1) Eastern Cooperative Oncology Group (ECOG), (2) Mobility derived digital biomarkers of frailty (MBF), and (3) a combination of ECOG and MBF. The parameters for MBF were chosen based on their significant differences and larger effect sizes between the resilient and non-resilient groups. Models incorporating MBF aimed to determine the optimal number of days needed to distinguish non-resilient cases from resilient ones as early as possible in post-chemotherapy initiation, while achieving an area under the curve (AUC) greater than 0.80.

The Chemotherapy Resilience Index (CRI) was calculated using fitted probabilities from a logistic regression model, featuring a scale from 0 to 1, where higher values indicate poorer resilience to chemotherapy. This model, derived using the maximum likelihood estimation (MLE) method^42^, was designed to optimally differentiate between resilient and non-resilient participants. To construct the model, we employed MBE metrics gathered during the shortest post-chemotherapy initiation time window that could effectively distinguish non-resilient cases from resilient ones with an AUC of 0.80 or greater. The aim was to develop a singular score reflecting the likelihood of poor resilience to chemotherapy as early as possible after the initiation of treatment. In assessing the predictive performance of CRI, in addition to sensitivity and specificity, we calculated the Positive Predictive Value (PPV) as shown in Eq. (1), using the optimal cut-point threshold determined by AUC.

1 $${\text{PPV}}= \frac{{\text{TP}}}{{\text{TP}}+{\text{FP}}}$$

To prevent an overestimation of the PPV, we computed the adjusted PPV using Eq. (2)^43^.

2 $$Adjusted PPV= \frac{Sensitivity \times Prevalence}{\left(Sensitivity \times Prevalence\right) + \{(1-Specificity)\times (1-Prevalence)\}}$$

The prevalence rate is derived from our dataset and represents the proportion of patients who experienced adverse reactions to chemotherapy (non-resilient) within our study sample.

All statistical analyses and machine learning models, including data extraction, plotting, and model fitting, were performed using MATLAB. (MathWorks Inc., Natick, MA, USA).

## Results

All Veterans admitted to the MDVAMC oncology clinic for chemotherapy treatment were considered as potential participants. The data were collected during two-time intervals: the first cohort between September 9, 2019, and October 16, 2019, and the second cohort from April 7, 2021, to April 12, 2022. However, recruitment for the second cohort experienced a pause of approximately six months due to the COVID-19 pandemic. Despite this, both cohorts followed the same protocol. Of these, thirty-nine Veterans [Age = 65.9 ± 13.9, BMI = 25.9 ± 5.4, 67% male, ECOG-PS: ECOG(0) = 33%, ECOG(1) = 41%, ECOG(2) = 18%] met the study's specific inclusion and exclusion criteria and consented to participate in the study. Among them, only one refused to wear the pendant sensor for 14 continuous days after giving consent, resulting in a 97% protocol feasibility rate for the use of the pendant sensor. The remaining thirty-eight participants completed the 14-day period and self-reported wearing the sensor continuously without removal. However, due to technical issues such as corrupted sensor memory and sensor battery failure that resulted in data not being recorded during the first two weeks, data from 11 participants had to be excluded. This resulted in a total of 27 valid samples. Among these, 14 participants (52%) completed a four-week chemotherapy cycle without encountering any adverse events during the cycle and survived up to six months post initiation, leading to their classification as resilient. The remaining thirteen participants (48%) were classified as non-resilient, having experienced one or more adverse events such as dose reduction or treatment discontinuation (n = 7, 54%), unplanned hospitalization (n = 8, 62%) within 4 weeks, or death within the first six months of therapy (n = 11, 85%). Baseline demographics and clinical characteristics are summarized in Table 2. Upon comparison, no significant differences were observed between the resilient and non-resilient groups in terms of demographics and comorbidities except the BMI and depression.

**Table 2:** Overall baseline demographics, clinical characteristics, and motor capacity information of the resilient and non-resilient group.

	Resilient (n = 14)	Non-resilient (n = 13)	*p*-value	Effect size (Cohen’s *d*)
Demographics				
Age, years	64.6 ± 12.9	67.2 ± 15.3	0.47	0.18
Sex (male), %	57.1%	76.9%	0.3	0.41
Body mass index, kg/m^2^	27.9 ± 6.4	23.8 ± 3.1	0.04*	0.82
Clinical characteristics				
ECOG = 0, %	57.1%	16.7%	0.02*	0.98
ECOG = 1, %	35.7%	50%		
ECOG = 2, %	7.1%	33.3%		
GI cancer	64%	92%	0.12	0.44
Lung cancer	14%	0%		
Breast cancer	14%	0%		
Hematologic cancer	7%	8%		
Stage 1	7%	0%	0.16	0.56
Stage 2	14%	17%		
Stage 3	21%	0%		
Stage 4	57%	83%		
Frailty index (TSFI), score	0.22 ± 0.08	0.25 ± 0.08	0.51	0.41
Depression, %	35.7%	0%	0.02*	1
Diabetes, %	50%	30.8%	0.33	0.38
High blood pressure, %	57.1%	61.5%	0.84	0.09
Stroke, %	0%	15.4%	0.15	0.59
Sleep problem, %	14.3%	23.1%	0.59	0.22

Values are presented as mean ± standard deviation (SD) or n (%).

*Significant difference between groups.

**Figure 2:**
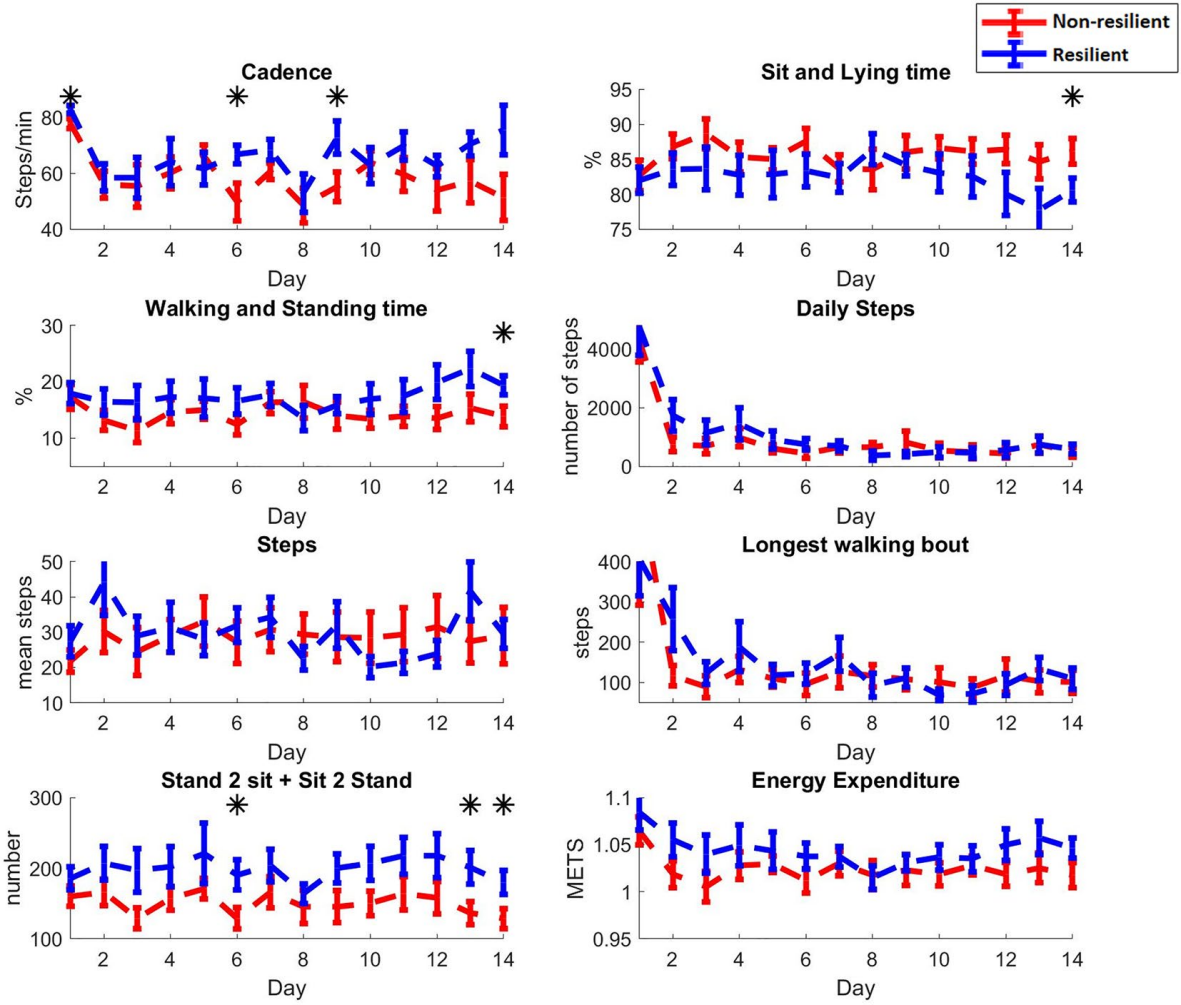
Sensor output metrics for a resilient (blue) and non-resilient (red) participants. The mean physical activity parameters between the groups are shown with error bar (standard error) for 2 weeks of recorded activity. The asterisk (*) sign is used to represent significant difference in parameters for the corresponding day between resilient and non-resilient participants.

Figure 2 illustrates the patterns of physical activity immediately following the initiation of the first chemotherapy cycle, over a duration of 14 days leading up to the subsequent cycle. Generally, visual observation of these patterns shows a decline in functional performance for both groups, with the most noticeable decline observed on either day 2 or 3 post-chemotherapy. However, the resilient group begins to recover from day 3 and reaches stable values for most parameters by around day 6. In line with our hypotheses, changes in frailty phenotypes like slowness (cadence) and weakness (postural transition) significantly predicted chemotherapy non-resilience cases, exhibiting large effect sizes (d = 0.86–0.88, p < 0.03). However, variations in inactivity and exhaustion, despite being observed in both groups, did not predict non-resilience in our sample (also shown in Supplemetary Table 1).

**Figure 3:**
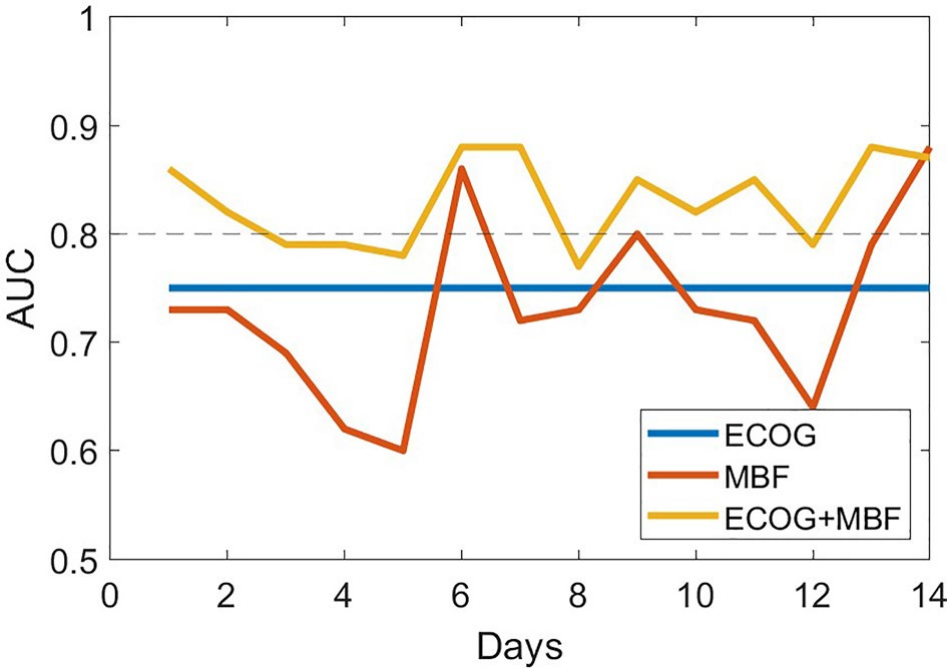
Area under curve (AUC) of the fitted logistic regression model for 3 different models shows the model that uses both ECOG and MBF can distinguish the resilient vs non-resilient group better than other models.

Based on regression analysis, we determined that the earliest day post-chemotherapy initiation capable of distinguishing between groups with an AUC greater than 0.80 is day 6. On this day, the model incorporating digital biomarkers of frailty achieved an AUC of 0.86, a notably superior performance compared to the model using only ECOG, which reached an AUC of 0.75. Additionally, the combination of ECOG with digital biomarkers of frailty slightly improved the model's performance, yielding an AUC of 0.88 in predicting the non-resilient group, as illustrated in Fig. 3 and Supplementary Figure 1.

**Figure 4:**
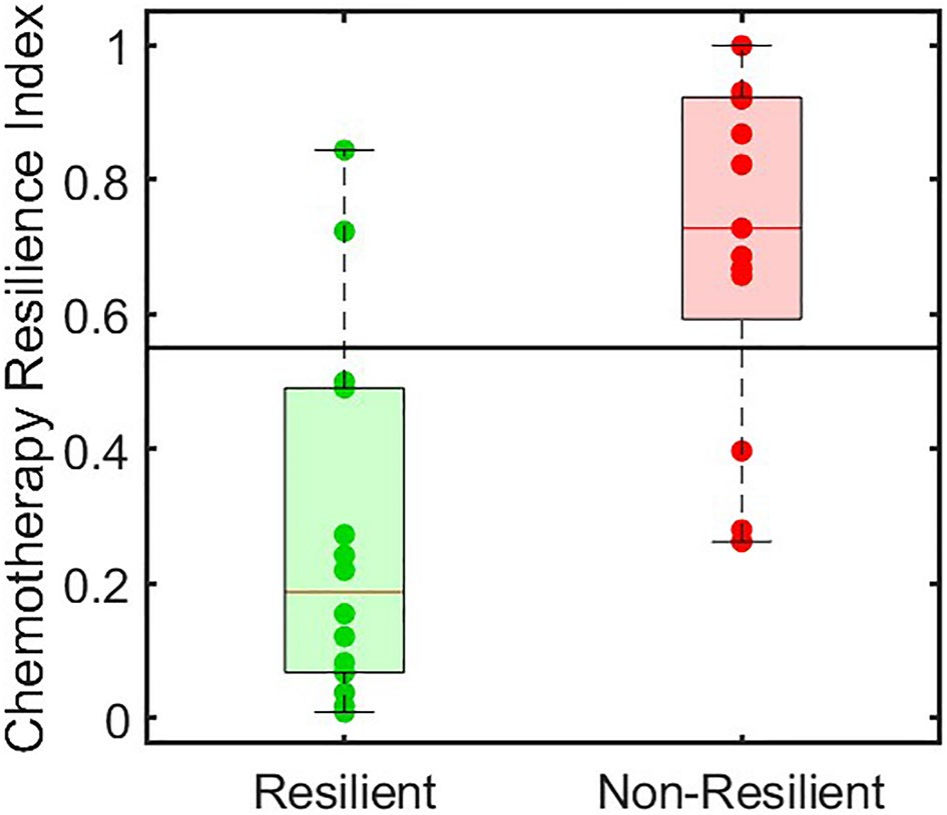
Chemotherapy resilience index (CRI ≥ 0.54) calculated from the MBF and ECOG can distinguish the patient with resilience significantly (p = 0.0007, effect size = 1.67).

The Chemotherapy Resilience Index (CRI) was calculated using a model that combines digital biomarkers of frailty, including cadence and postural transitions, measured on Day 6 post-chemotherapy initiation, along with ECOG values. This index is normalized on a scale from 0 to 1, with higher values indicating greater severity in the lack of resilience to chemotherapy. Using this index, we successfully distinguished between groups with a very large effect size, as demonstrated in Fig. 4 (CRI = 0.2 ± 0.27 in the resilient group vs. 0.7 ± 0.26 in the non-resilient group, p < 0.001, Cohen’s d = 1.67). The optimal cut-off point for the CRI was established at 0.54. At this threshold, we achieved a sensitivity of 77%, a specificity of 86%, and an adjusted PPV of 83% for distinguishing non-resilient cases from resilient ones on day 6 post-chemotherapy initiation.

## Discussion

In this observational study, we investigated whether frailty phenotypes derived from physical activity were associated with chemotherapy resilience among cancer patients. Our results support the hypothesis that analyzing the phenotypes of frailty can help differentiate individuals who are resilient and who are not resilient. Specifically, slowness and weakness phenotypes achieved large effect size range in distinguishing the two groups; however, variations in inactivity and exhaustion, despite being observed in both groups, did not distinguish non-resilience in our sample. Additionally, our study indicates that the earliest day post-chemotherapy initiation for effectively predicting the non-resilient group with an AUC greater than 0.80 is day 6 post-therapy. On this day, our model, utilizing digital biomarkers of frailty measured via a pendant sensor combining with ECOG, predicts non-resilience with an AUC of 0.88. This timing could offer a sufficient window for timely interventions or decision-making prior to the next therapy cycle, which is typically scheduled on a bi-weekly basis.

Interestingly, the patterns of physical activity following the initiation of chemotherapy indicate a decline in most functional metrics for both groups, particularly in cadence, with the largest decrease observed on days 2 or 3 post-therapy initiation. The functional performance metrics in the resilient group begin to rebound from day 3 and almost return to pre-therapy levels by day 6. In contrast, the non-resilient group either fails to recover in some metrics, recovers over a longer time period, or in some cases, further deteriorates after day 6. This pattern may explain why our model, which distinguishes between the groups, shows its strongest performance on days 6 and 7, with less stable results thereafter.

The link between frailty and non-resilience to chemotherapy in older adults is a critical area of research, with multiple established methods such as the Liver Frailty Index and Fried Frailty Index (FFI) being used to predict a patient’s capacity to cope with treatment^44–49^. Traditional screening methods for detecting frailty, while informative, are often subjective, necessitate in-person administration, and demand considerable resources—posing substantial barriers for those who are frail and may find it challenging to visit clinics regularly. Additionally, traditional methods often rely on a single snapshot assessment of functional capacity, typically conducted in a clinical setting prior to the initiation of chemotherapy. This approach, however, lacks the temporal resolution necessary to track potential functional deterioration due to chemotherapy and subsequent recovery patterns. As defined by Hale et al.^50^, resilience is the capacity to recover quickly from difficulties or adapt effectively in the face of stress or trauma. In our study, we propose that continuous monitoring of digital biomarkers of frailty, such as a decrease in cadence (a surrogate for slowness^24^) and a reduction in the total daily number of postural transitions (a surrogate for weakness^24^), derived from daily physical activity, may aid in determining and quantifying resilience to chemotherapy. This method evaluates the extent of functional performance deterioration in response to chemotherapy and the speed of returning to baseline levels established prior to chemotherapy initiation. Our study may also pave the way for deploying remote patient monitoring solutions as a practical and objective tool for remotely screening resilience to chemotherapy and potentially assessing the level of toxicity accumulation post-therapy. This offers significant benefits over conventional techniques that rely on assessments under supervised conditions, often in oncology clinics. This approach not only mitigates the logistical and resource-intensive aspects of traditional screenings but also introduces a critical element of temporal resolution to the monitoring process. With this added time resolution, our study reveals that remote monitoring of physical activities and metrics, such as a decline in cadence and a reduction in postural transition numbers (daily number of sit-to-stand and stand-to-sit movements), can identify individuals with reduced resilience to chemotherapy as early as 6 days after treatment commencement. This method has an adjusted 83% PPV in predicting those who develop major adverse events up to 6 months post-chemotherapy initiation. Further research is necessary to determine if this timeframe allows for timely intervention. Nonetheless, we believe this information can significantly enhance personalized decision-making for subsequent chemotherapy cycles, potentially guiding oncologists to adjust treatment strategies. This may include altering chemotherapy agents, reducing doses, or discontinuing therapy early to prevent severe adverse events like unplanned hospitalizations or death.

The prominence of the decline in cadence and the parameters related to postural transitions within this context can be attributed to their status as indicative markers of slowness and weakness which are recognized hallmarks of frailty^51^. These findings are consistent with studies which evaluated the association between specific components of FFI and chemotherapy toxicity and found slowness (gait speed) and weakness (grip strength) to be significantly associated with toxicity^45,52^. Another study demonstrated the significant association between prolonged Timed Up and Go with chemotherapy toxicity as well which adds value to the role of physical activity parameters as predictors of chemotherapy-induced toxicity^53^ and it is also comprehensively discussed in a systematic review and meta-analysis that physical activity decreases the severity of side effects of cancer treatment besides reduction in risk of cancer recurrence and death^54^. This alignment with prior research further strengthens the premise that our observations are rooted in a well-established framework.

One of the exploratory objectives of this study was to determine the earliest post-chemotherapy time point at which poor resilience can be predicted. Early identification of poor resilience to chemotherapy could inform decisions about subsequent treatment cycles. While chemotherapy cycles vary significantly depending on cancer type, specific drugs used, treatment goals (curative, control, or palliative), and the individual patient’s health and response to therapy, a common schedule is bi-weekly^55^. In our study, all participants followed a bi-weekly schedule, and we tracked changes in physical activity patterns during the 14 days leading up to the next cycle. To identify the earliest post-chemotherapy time point, we applied regression model analysis to our time series data, seeking the earliest time point where groups could be distinguished with an AUC greater than 0.80. We found that as early as 6 days post-chemotherapy, we could differentiate between groups with an AUC greater than 0.80. Initially, both groups, regardless of resilience level, showed a decline in functional capacity, making the first few days less ideal for distinguishing those with poor resilience. However, we observed that the resilient group’s functional capacity quickly returned to baseline values before the initiation of chemotherapy, whereas the recovery in the non-resilient group required more days. Therefore, around the mid-point of the cycle (days 6 or 7), appears to be the optimal time to distinguish recovery patterns between the two groups. While extending the observation period beyond the midpoint may increase the detection accuracy for some functional capacity metrics, the reliability of the results could be compromised. This is because individuals in the non-resilient group may exhibit some level of recovery over a longer time window, thus diminishing the differences between groups over extended intervals. This phenomenon might explain the high variation in estimated AUC values observed after day 6. However, these speculations need to be validated in future studies with larger sample sizes.

A significant limitation of our study in determining the optimal time point for detecting non-resilience was the necessity of using the entire dataset for model fitting, due to the small sample size, which may lead to inaccuracies. Future studies should remedy this by dividing the dataset into separate portions for model fitting and testing. Nevertheless, with the assumption that 6 days post-chemotherapy is an optimum point to identify non-resilient cases, we introduced the concept of a Chemotherapy Resilience Index (CRI). This index, ranging from 0 to 1, where higher values indicate an increased likelihood of experiencing major adverse events, was calculated 6 days after the initiation of therapy. The CRI demonstrated a remarkable ability to distinguish between non-resilient and resilient groups, indicating a very large effect size (d = 1.67). These results highlight the potential of leveraging digital biomarkers from remote monitoring systems to enhance predictions of treatment resilience in cancer patients.

Our study revealed that there were no significant demographic or comorbidity differences between the resilient and non-resilient patient groups except BMI and depression, highlighting the significant role of frailty biomarkers in assessing chemotherapy-induced resilience. BMI has positive correlation with resilience (higher BMI is linked to a lower risk of non-resilience which is align with similar studies that patients with higher BMI experienced less severe chemotherapy-induced toxicity in particular hematologic and gastrointestinal events and hospital admissions^56–58^). One possible reason could be the different pharmacokinetics of antineoplastic drugs in the bodies of overweight individuals. There is published evidence showing that some drugs may lead to reduced levels in obese individuals^59^. It has also been illustrated that lower BMI and specifically sarcopenia, as a characteristic syndrome of progressive and generalized skeletal loss^60^, is significantly associated with frailty^61^ and pre-therapeutic sarcopenia is found to have a predictive value for chemotherapy-induced toxicity^62^. So, considering lower BMI as an alien with frailty for predicting non-resiliency is well demonstrated in our study and is consistent with the aforementioned investigations. The unexpected finding that the rate of depression was significantly higher in the resilient group (35.7%) compared to the non-resilient group (0%) challenges prevailing assumptions about the interplay of mental health and resilience in cancer patients^9^. This could imply that physical frailty may be a more significant predictor of resilience to chemotherapy than the presence of depression. However, given the small sample size of our study, we must consider the possibility that our results may not sufficiently capture the role of depression. Consequently, these preliminary findings are speculative, and further investigation with a larger cohort is essential to substantiate the relationship between depression and chemotherapy resilience.

Our predictive model built on the combination of these physical activity patterns could identify the non-resilient group with an AUC of 0.86, which surpassed the predictive capacity of the ECOG-PS assessment (AUC = 0.75). The integration of physical activity digital biomarkers and the ECOG-PS assessment slightly improved the AUC to 0.88 which was an indicator of better efficacy of combining these items instead of using them separately. Additionally, our results indicate that the model combining physical activity and ECOG outperforms in terms of differentiation capability, surpassing the clinically accepted threshold of 0.80 AUC^63^. In contrast, the ECOG alone does not meet this benchmark. This lends credence to our hypothesis that integrating digital metrics with in-clinic evaluations enhances the accuracy in identifying individuals who experienced chemotherapy-related adverse events from those who did not.

In our study, we employed a pendant sensor to remotely monitor physical activities and indicators of frailty such as slowness and weakness. The choice of a pendant sensor likely contributed to the high adherence and acceptability observed in our target population. Acceptability is critical, as the design and ergonomics of wearable devices significantly influence their potential for continuous health monitoring and early prediction of resilience to cancer treatment toxicity. The integration success of monitoring technology relies on the patients' comfort and their willingness to wear the devices consistently, particularly during the vital initial therapy phase. Cumbersome sensors that interfere with daily life are less likely to be accepted^34,64^. Wrist-worn sensors are effective for logging daily steps and periods of inactivity, yet they may lack the sensitivity needed to capture intricate physical activity patterns crucial for an in-depth evaluation of functional performance^64,65^. These details include cadence and postural transitions, which have been identified as more indicative of chemotherapy resilience than mere step count. Thus, the strategic placement of the sensor is as important as its function. Moreover, the discretion offered by a sensor that can be concealed during daily activities likely enhances its acceptance, as individuals may prefer to keep wrist space available for personal accessories rather than medical devices. Our study's findings support the feasibility and high acceptability of the pendant sensor-based system for remote patient monitoring, with an acceptance rate of 97%, surpassing that of similar studies (83%)^66^. This success may be attributed to the sensor's unobtrusive and practical design, underlining the importance of considering patient convenience in wearable health technology.

This pilot study, while insightful, comes with several limitations. Being a single-center feasibility study, our sample was both small and homogenous. This homogeneity might account for the lack of significant differences in demographics and comorbidities between resilience groups. Furthermore, the majority of our sample was white and male, which represent the demographics of Veterans. Additionally, due to the small sample size, we used the entire dataset for our model fitting process, instead of partitioning it into separate portions for fitting and testing the models. Given these factors, there's a pressing need to validate our findings using a larger, more diverse sample that truly represents the demographic spread of individuals undergoing chemotherapy. It's important to note that Veterans receiving chemotherapy have distinct characteristics; their comorbidities, health behaviors, and access to healthcare might differ from the broader population. By pinpointing specific challenges and opportunities related to predicting and addressing cancer-related toxicity adverse events in this group, beyond just ECOG and physical activity metrics, we can tailor and enhance care for Veterans diagnosed with cancer. Future research should prioritize the development of remote patient monitoring systems capable of utilizing digital biomarkers and predictive analytics to anticipate the occurrence or likelihood of adverse events. Furthermore, it is imperative to examine the perspectives of clinicians and patients regarding the trade-off involved in integrating physical activity monitoring into such systems, preferably through a comprehensive investigation involving a larger sample size.

## Conclusion

This study establishes a proof of concept for the remote monitoring of daily physical activities for the continuous monitoring of chemotherapy resilience in cancer patients. Our observations suggest that by continuously monitoring physical activity, as the digital biomarkers of frailty phenotypes, over a 14-day period using a pendant sensor and combining this data with in-clinic ECOG assessments, we can identify patients who might be at risk of poor response to chemotherapy. This knowledge has the potential to equip both oncologists and patients with essential information, enabling them to make informed decisions regarding chemotherapy treatment adjustments. Moreover, this insight can pave the way for potential interventions like structured exercise programs or nutritional management strategies, aiming to bolster patient resilience and minimize adverse events related to treatment.

It's noteworthy to emphasize that digital biomarkers linked to slowness and weakness appear to be potent predictors of adverse events in cancer patients undergoing chemotherapy. These findings underscore the importance of a more in-depth exploration into the realm of digital biomarkers. However, it's essential to acknowledge that this study's limited sample size means that the observations need validation in broader and more diverse patient cohorts.

As we pivot towards practical implementation, capturing the perspectives of both patients and physicians regarding the integration of wearable technology into routine clinical practices becomes imperative. Ultimately, the introduction of these digital frailty biomarkers promises to revolutionize physical performance evaluations, seamlessly blending traditional clinical assessments with precise, objective digital data. The research confirms the effectiveness of a remote monitoring system that combines a pendant sensor with ECOG assessments. This has the potential to enhance patient monitoring and improve chemotherapy safety, pending validation in larger study samples.

### Supplementary Information

Supplementary Information.

Supplementary Figure 1.

Supplementary Table 1.

## Data Availability

The raw data supporting the conclusions of this study are not publicly accessible due to privacy concerns. However, processed de-identified data that do not compromise participant anonymity are available upon formal request to the senior author, Bijan Najafi.
