# Supplementary material for: Harnessing physical activity monitoring and digital biomarkers of frailty from pendant based wearables to predict chemotherapy resilience in veterans with cancer
Source: Sci Rep. 2024 Jan 31;14:2612. doi: 10.1038/s41598-024-53025-z (PMC10831115; doi:10.1038/s41598-024-53025-z)
Supplement: Supplementary file 1 — Supplementary Information. [file 41598_2024_53025_MOESM1_ESM.pdf]

Harnessing Physical Activity Monitoring and Digital Biomarkers of Frailty from Pendant Based Wearables to Predict Chemotherapy Resilience in Veterans with Cancer

Running Title: Digital Metrics of Chemotherapy Related Toxicity

Gozde Cay, PhD[1], Yvonne H Sada, MD, MPH[2], Mohammad Dehghan Rouzi, MS[1], Md Moin Uddin Atique, PhD[1], Naima Rodriguez[1], Mehrnaz Azarian, MD[1], MG Finco, PhD[1], Sarvari Yellapragada, MD[2], Bijan Najafi, PhD*[1]

Affiliations:
[1]Interdisciplinary Consortium on Advanced Motion Performance (iCAMP), Michael E. DeBakey Department of Surgery, Baylor College of Medicine, Houston, TX
[2]Michael E. DeBakey VA Medical Center, Houston, TX

ECOG Performance Status

These scales and criteria are used by doctors and researchers to assess how a patient's disease is progressing, assess how the disease affects the daily living abilities of the patient, and determine appropriate treatment and prognosis. They are included here for health care professionals to access.

ECOG PERFORMANCE STATUS*	
**Grade**	**ECOG**
0	Fully active, able to carry on all pre-disease performance without restriction
1	Restricted in physically strenuous activity but ambulatory and able to carry out work of a light or sedentary nature, e.g., light house work, office work
2	Ambulatory and capable of all selfcare but unable to carry out any work activities. Up and about more than 50% of waking hours
3	Capable of only limited selfcare, confined to bed or chair more than 50% of waking hours
4	Completely disabled. Cannot carry on any selfcare. Totally confined to bed or chair
5	Dead

* As published in Am. J. Clin. Oncol.:
Oken, M.M., Creech, R.H., Tormey, D.C., Horton, J., Davis, T.E., McFadden, E.T., Carbone, P.P.: Toxicity And Response Criteria Of The Eastern Cooperative Oncology Group. Am J Clin Oncol 5:649-655, 1982.
