# Supplementary material for: Harnessing physical activity monitoring and digital biomarkers of frailty from pendant based wearables to predict chemotherapy resilience in veterans with cancer
Source: Sci Rep. 2024 Jan 31;14:2612. doi: 10.1038/s41598-024-53025-z (PMC10831115; doi:10.1038/s41598-024-53025-z)
Supplement: Supplementary file 2 — Supplementary Figure 1. [file 41598_2024_53025_MOESM2_ESM.docx]

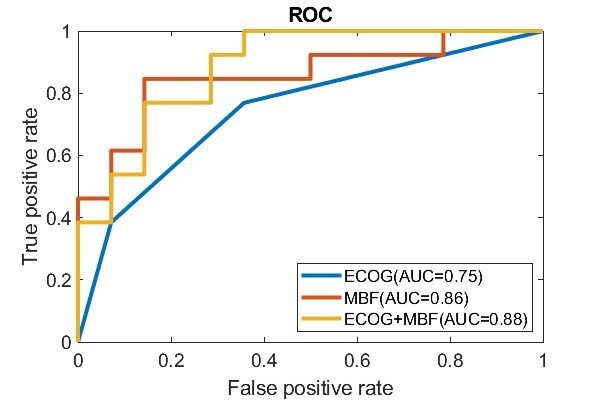


**Supplementary Figure:** ROC curve for the logistic regression model to distinguish resilient vs non-resilient population on the 6th day of the chemotherapy. The ROC for the three models, using ECOG or MBF or ECOG and MBF and the corresponding area under curve (AUC) represents the model fits best when ECOG and MBF are combined to distinguish the groups (AUC=0.88).
