# Supplementary material for: Harnessing physical activity monitoring and digital biomarkers of frailty from pendant based wearables to predict chemotherapy resilience in veterans with cancer
Source: Sci Rep. 2024 Jan 31;14:2612. doi: 10.1038/s41598-024-53025-z (PMC10831115; doi:10.1038/s41598-024-53025-z)
Supplement: Supplementary file 3 — Supplementary Table 1. [file 41598_2024_53025_MOESM3_ESM.docx]

Table 1: Univariate parameters between groups and their effect sizes at baseline, 6 days post-chemotherapy and endpoint (14-days)

|  | Resilient (n=14) | | | Non-resilient (n=13) | | | p-value | | | Effect Size (Cohen's d) | | |
| --- | --- | --- | --- | --- | --- | --- | --- | --- | --- | --- | --- | --- |
| Parameter | Baseline | 6th day | End point | Baseline | 6th day | End point | Baseline | 6th day | End point | Baseline | 6th day | End point |
| Cadence | 82.8±1.4 | 66.7±3.3 | 75.5±8.8 | 77.8±1.8 | 49.8±6.7 | 51.4±8.2 | 0.038 | **0.034** | 0.062 | 0.85 | 0.87 | 0.75 |
| Sit and Lying time | 82.0±1.9 | 83.4±2.3 | 80.6±1.7 | 82.7±2.2 | 87.6±1.8 | 86.1±1.8 | 0.819 | 0.177 | **0.039** | 0.09 | 0.53 | 0.84 |
| Walking and Standing time | 18.0±1.9 | 16.6±2.3 | 19.4±1.7 | 17.3±2.2 | 12.4±1.8 | 13.9±1.8 | 0.82 | 0.177 | **0.039** | 0.09 | 0.53 | 0.84 |
| Daily Steps | 4743.6±962.4 | 753.6±195.2 | 593.1±161.1 | 4284.8±728.8 | 447.6±168.3 | 481.1±163.2 | 0.714 | 0.256 | 0.636 | 0.14 | 0.45 | 0.18 |
| Steps | 27.4±4.4 | 32.0±4.9 | 29.5±4.1 | 21.8±3.1 | 27.2±6.0 | 29.0±8.0 | 0.32 | 0.544 | 0.956 | 0.39 | 0.24 | 0.02 |
| Longest walking bout | 405.9±90.7 | 122.4±26.0 | 109.6±26.4 | 585.9±293.7 | 95.8±27.9 | 102.0±28.7 | 0.564 | 0.501 | 0.849 | 0.23 | 0.26 | 0.07 |
| Stand 2 sit + Sit 2 Stand | 185.6±16.3 | 190.6±21.4 | 179.9±17.0 | 160.4±14.3 | 129.2±15.2 | 128.5±14.1 | 0.265 | **0.031** | **0.032** | 0.44 | 0.88 | 0.88 |
| Energy Expenditure | 1.1±0.0 | 1.0±0.0 | 1.0±0.0 | 1.1±0.0 | 1.0±0.0 | 1.0±0.0 | 0.429 | 0.186 | 0.11 | 0.31 | 0.52 | 0.64 |
